# Supplementary material for: Dynamic transcriptomic profiles of zebrafish gills in response to zinc supplementation
Source: BMC Genomics. 2010 Oct 11;11:553. doi: 10.1186/1471-2164-11-553 (PMC3091702; doi:10.1186/1471-2164-11-553)
Supplement: Additional file 2 — Interactive Direct Interaction Network representing the molecular interactions between zinc, copper, iron, calcium and proteins encoded by transcripts changed by zinc supplementation. Mini web-site containing index.html and hyperlinked pages in subdirectory describing a Direct Interaction Network automatically generated based on curated interactions contained within the proprietary PathwayArchitect database. Ovals represent proteins and the circles symbolize metal ions. Objects are coloured by their abundance in zebrafish at the time-point they were significantly different from the control is a scale from -4 fold (dark green) to +4 fold (dark red). Where significant differences were found at more than one time-point, the colour overlay shows expression at the first instance. Dark blue squares denote 'binding', and light blue squares 'expression'; green squares stand for 'regulation', green diamonds for 'metabolism', and green circles for 'promoter binding'. Arrow heads indicate directionality of the interaction where annotated. All nodes and edges can be further interrogated by selecting the relative area of the image. [file 1471-2164-11-553-S2.zip › PathwayArchitect Zn xs DIN/401678.html]

# REGULATION:

|  |  |
| --- | --- |
| Type | REGULATION |
| Effect | Positive |


---

|  |  |
| --- | --- |
| Score | 0 |


---

|  |  |
| --- | --- |
| Reference Count | 5 |


---

|  |  |
| --- | --- |
| Mechanism | Unknown |


---

|  |  |
| --- | --- |
| Reference:0 || Sentence | "Since previous studies have demonstrated that components of the AP1 transcription factor can modulate glucocorticoid receptor activity in other systems, we examined the regulation of the proto-oncogenes c-fos and c-jun in response to CRF." |
| PMID | 8375573 |
| Year | 1993 |
| Species | Rat |
| Journal | Mol Cell Endocrinol |
| RefScore | 2 |
| Source | PArchNLP |
  |
|  |  |


---

|  |  |
| --- | --- |
 Reference:1 || Sentence | "Gel shift analysis demonstrated that CRF caused a sustained increase in AP-1 DNA binding to both a canonical AP-1 element as well as to the POMC exon-1 AP-1 site." |
| PMID | 8793851 |
| Year | 1996 |
| Species | Mouse |
| Journal | Mol Cell Endocrinol |
| RefScore | 1 |
| Source | PArchNLP |
  ||  |  |


---

|  |  |
| --- | --- |
 Reference:2 || Sentence | "Although DEX treatment of AtT-20 cells did not affect AP-1 DNA binding capacity of nuclear extracts, DEX pretreatment blunted the stimulation of AP-1 binding in response to CRF." |
| PMID | 7894465 |
| Year | 1994 |
| Species | Mouse |
| Journal | J Neuroendocrinol |
| RefScore | 2 |
| Source | PArchNLP |
  ||  |  |


---

|  |  |
| --- | --- |
 Reference:3 || Sentence | "High levels of DEX treated extracts led to a relative repression of CRF-induced AP-1 binding, suggesting that ligand-activated GR may lower available AP-1 levels by direct protein: protein interaction." |
| PMID | 7894465 |
| Year | 1994 |
| Species | Mouse |
| Journal | J Neuroendocrinol |
| RefScore | 1 |
| Source | PArchNLP |
  ||  |  |


---

|  |  |
| --- | --- |
 Reference:4 || Sentence | CRH also increased AP-1 binding activity, cell granularity, cytokeratin 1 and involucrin expression, and inhibited cytokeratin 14 expression. |
| PMID | 15787816 |
| Year | 2005 |
| Species | Human |
| Journal | Br J Dermatol |
| RefScore | 1 |
| Source | PArchNLP |
  |


---

|  |  |
| --- | --- |
